# Anticholinergic Burden and Dry Mouth Problems Among Older Adults (≥ 50 Years) Receiving Dental Care—A Retrospective, Cross‐Sectional Analysis

**DOI:** 10.1002/cre2.70009

**Published:** 2024-11-05

**Authors:** Christoph Matthias Schoppmeier, Isabel Deeg, Michael Jochen Wicht, Anna Greta Barbe

**Affiliations:** ^1^ Polyclinic for Operative Dentistry and Periodontology Faculty of Medicine and University Hospital Cologne, University of Cologne Cologne Germany

**Keywords:** elderly, German anticholinergic burden score, hyposalivation, xerostomia

## Abstract

**Objectives:**

Anticholinergics cause dry mouth and are highly relevant for dentists, but little is known about the relationships between intake and the occurrence of subjective and objective dry mouth with age. The German anticholinergic burden score (GACB) is a novel anticholinergic score that re‐evaluates medications, particularly, those with classification discrepancies.

**Material and Methods:**

We retrospectively investigated the GACB in older patients receiving dental care, evaluated whether GACB is related to xerostomia and unstimulated salivary secretion, and determined the influence of increasing age (beginning at 50 years of age). The GACB score quantified cumulative anticholinergic effects: 0 for no effect, 1 for possible, 2 for moderate, and 3 for strong. Cross‐sectional data in patients ≥ 50 years were collected, including xerostomia with the visual analog scale, unstimulated salivary flow rates, and the GACB scores.

**Results:**

Among 172 patients (mean age 65.67 ± 9.51 years), 23.8% had a GACB score ≥ 1. A moderate negative correlation was observed between GACB and unstimulated salivary flow rates ($\overset{®}{r}$ = −0.51). Patients with GACB ≥ 1 had fewer teeth (mean 21.76 ± 5.41) than those with GACB = 0 (24.07 ± 5.57). Moreover, unstimulated hyposalivation was observed in 61.0% with GACB ≥ 1 versus 6.8% with GACB = 0 (*p* < 0.001). Escalating chronic systemic conditions and prescribed medications were recorded with increasing age; those aged 76–80 years had the highest burden.

**Conclusions:**

The GACB quickly and reliably assesses anticholinergic exposure and risks for oral health in older patients. Routine use in those aged ≥ 50 years could enable early identification of risks and initiation of preventive dental measures.

**Trial Registration:**

German Registry for Clinical Trials: DRKS00032877 (https://www.germanctr.de; date of registration: 17.10.2023).

## 1 Introduction

Increasing medication usage with age is highly relevant in dentistry. Polypharmacy, defined as the intake of five or more medications (Lee et al. 2020; van den Akker et al. 2019), significantly contributes to hyposalivation and xerostomia in older adults and complicates dental diagnostic and treatment (Masnoon et al. 2017; Schoppmeier et al. 2022). Anticholinergics, such as benztropine and trihexyphenidyl for Parkinson's disease or ipratropium and tiotropium for chronic obstructive pulmonary disease, inhibit acetylcholine action at muscarinic acetylcholine receptors on salivary gland cells (Proctor 2016), reducing saliva flow and causing dry mouth (Nishtala, Salahudeen, and Hilmer 2016). Other medications, including opioids and antidepressants, also induce anticholinergic effects, with older individuals being particularly susceptible (Salahudeen, Duffull, and Nishtala 2015). The risk increases with multimorbidity, which affects over half of older people (Kaufman et al. 2002). This chronic medication use, beginning earlier in life, can lead to neurological side effects like hyposalivation, delirium, increased fall risk, blurred vision, urinary retention, tremor, and hallucinations, severely impacting the quality of life and dental care (Andrade 2019; Byrne et al. 2018; Campbell et al. 2016; Green et al. 2019; Mintzer and Burns 2000; Salahudeen, Duffull, and Nishtala 2015). Prolonged use of anticholinergic medications in older adults may significantly increase the risk of cognitive decline due to their cumulative effect over time (Pieper et al. 2020). The combined anticholinergic effect of several medications leads to an anticholinergic burden (ACB); older patients are more susceptible due to reduced metabolic capacity and slower drug elimination (Feinberg 1993). Medication‐induced dry mouth in older adults can adversely affect oral hygiene, reduce therapeutic options in dental practice, decrease saliva flow, increase the risk of (root) caries and microbial changes, and lower oral health‐related quality of life (Barbe 2018). Routine dental care does not currently assess the anticholinergic risk profile associated with medication use, but identifying this risk before oral health deteriorates is crucial for prevention‐oriented care (Barbe and Noack 2021). The primary focus in dentistry should be on assessing dry mouth risk, which directly impacts therapy planning. Various lists are available to calculate the cumulative effect of anticholinergic drugs, resulting in a score that classifies the risk based on the total number of drugs taken (Ali et al. 2020).

Internationally recognized scoring systems, such as the Anticholinergic Drug Scale (ADS) and the global ACB Scale, evaluate and classify medications based on their anticholinergic potential (Campbell et al. 2013; Carnahan et al. 2006). A new ACB score specific to the German market (GACB) was introduced by Kiesel et al. to reduce the number of potentially inappropriate medications prescribed to older patients (Kiesel, Hopf, and Drey 2018). The group scored 504 drugs available in Germany, categorizing them as having no, weak, moderate, or strong anticholinergic effects. Although the GACB is tailored to the German medication market for direct clinical relevance, its structural similarity to international systems facilitates comparative analysis. To our knowledge, no study has as yet investigated the GACB score in dental patients at risk, defined as patients aged 50 years and older. Additionally, no data have evaluated the association between GACB scores and both objective and subjective measures of dry mouth, as well as clinical parameters of oral health. It is also unclear to what extent the GACB score represents a risk for dry mouth. This knowledge is essential to recommend using the GACB score in dental practice for dry mouth risk assessment. Thus, our retrospective, cross‐sectional study aimed to investigate the GACB among people aged ≥ 50 years (a group known to be at greater risk of polypharmacy with increasing age) receiving dental care. We also investigated whether the ACB of drugs was related to xerostomia and unstimulated salivary secretion among this study sample, as well as the influence of increasing age.

## 2 Methods

### 2.1 Study Sample

The retrospective cross‐sectional study approval was obtained from the Ethics Committee of the University of Cologne, Germany (23‐1062‐retro, approval date 06‐02‐2023). The research was performed in accordance with the Declaration of Helsinki and is reported according to STROBE guidelines (von Elm et al. 2014). Data were retrospectively collected from adults (aged ≥ 50 years) who received dental care from the student classes at the Department of Operative Dentistry and Periodontology, Cologne, Germany, between October 2022 and March 2023. Dental students, under the supervision of experienced clinical instructors calibrated for the assessed clinical data, conducted a comprehensive anamnestic and clinical investigation. During their preclinical education in the fifth and sixth semesters, all dental students are required to undergo comprehensive training and education that includes an objective structured clinical examination that focuses on taking medical histories and diagnoses. Each patient examined by these students was required to provide a medication list, confirmed by their general practitioner, detailing all regularly taken medications and a comprehensive list of chronic systemic diseases. This list was subsequently verified against the electronic medication plans (eMPs) on the electronic health card. Additionally, further clinical parameters were recorded, including DMFT (Decayed, Missing, and Filled Teeth) scores, the Periodontal Screening Index (PSI), unstimulated salivary flow rate (USFR), and an evaluation of subjectively perceived dry mouth using a validated visual analog scale (VAS) (Dirix et al. 2008). As our study involved a retrospective analysis of these existing medical records, collected during routine dental care, individual patient consent was deemed unnecessary by the Ethics Committee. The data sets analyzed and referenced throughout this investigation are available upon request from the corresponding author.

### 2.2 Clinical Parameters

#### 2.2.1 DMFT

For each patient, the number of decayed (D), missing (M), or filled (F) teeth (T) was recorded and the DMFT was calculated (WHO 1997). DMFT values are collected and processed as a standard procedure during every patient examination and hence are reported automatically. In our retrospective analysis, the reasons for missing teeth, that is, missing due to periodontitis, caries, and/or orthodontic treatment, were not available.

#### 2.2.2 PSI

To evaluate the periodontal health status, we collected the PSI data, which had been evaluated using a PCP UNC 15 probe (Landry and Jean 2002). Teeth were divided into sextants, and each tooth was examined at six distinct points: mesial, central, and distal on both the buccal and the lingual/palatal sides. The index uses codes ranging from 0 to 4, with the highest code of any sextant recorded as the overall PSI value. A code of 0 indicates a healthy periodontal condition with a probing depth of less than 3.5 mm and no bleeding on probing, dental calculus, or any inadequate restoration margins present. Codes 1 and 2 are indicative of gingivitis: with a probing depth of less than 3.5 mm, Code 1 is characterized by bleeding on probing, whereas Code 2 also identifies the presence of dental calculus or inadequate restoration margins. Code 3 corresponds to moderate periodontitis with a probing depth between 3.5 and 5.5 mm. Code 4 signifies severe periodontitis, where the probing depth exceeds 5.5 mm. The highest recorded PSI value was used to classify the severity of periodontitis in the patients examined.

#### 2.2.3 USFR

All saliva collections (a routine procedure for patients ≥ 50 years) took place during the student courses at the Department of Operative Dentistry and Periodontology, Cologne, Germany, between 8:00 and 11 a.m. to avoid circadian fluctuations (Dawes and Ong 1973). Patients did not consume any food or drinks, smoke, use mouthwash, rinse out their mouth, or perform oral hygiene for at least 30 min before the examination. A draining method had been used to measure the USFR (Navazesh 1993). Patients were asked to sit straight in a dental chair, swallow, bend forward, and asked to hold their mouth open and remain still, letting the saliva drip into a disposable pre‐weighted cup held to the lower lips for 5 min (Stenbäck et al. 2023). Volumes (mL) were determined. Salivary volume measurements were obtained through gravimetric analysis, utilizing Luer slip syringes (BD Discardit II; Becton Dickinson), an accurately calibrated analytical scale, and the weight of the saliva, based on a presumed specific gravity of 1.0. Salivary flow rates are reported in mL/min. Collection of unstimulated saliva was conducted with removable dentures taken out. Threshold values were defined as ≤ 0.1 mL/min (hyposalivation) and > 0.1 mL/min (normosalivation) for USFR (Villa et al. 2016).

#### 2.2.4 VAS

Patients had recorded their answers to the question “How dry is your mouth?” on a VAS (cm), from 0 = “not dry at all” to 10 = “no saliva at all,” which had been validated in a previous study (Dirix et al. 2008). Patients also gave their subjective overall assessment of dry mouth at different sites within the mouth, in response to the question “How dry does it feel at the palatal mucosa/buccal mucosa/anterior tongue/lower lip?.”

### 2.3 GACB

The GACB score was determined retrospectively using the method of Kiesel, Hopf, and Drey (2018), used to quantify the cumulative anticholinergic effect of medications taken by patients. It represents a further development of the ADS (Carnahan et al. 2006). Each medication is assigned to one of three categories, based on its anticholinergic potency:

- Score 0: drugs with no anticholinergic effect.
- Score 1: drugs with possible anticholinergic effects. These are drugs in which anticholinergic effects have been observed but do not always occur.
- Scores 2 and 3: drugs with definite anticholinergic effects. Drugs with a score of 2 have moderate anticholinergic effects, whereas drugs with a score of 3 have strong anticholinergic effects and are usually associated with a higher risk of side effects and adverse clinical events.

### 2.4 Sample Size

Due to the retrospective study design, no case number was defined. The case number presented here is based on the defined evaluation period from October 2022 to March 2023, when dry mouth diagnostics were routinely introduced at the University Hospital Cologne.

### 2.5 Statistical Analysis

Data were analyzed descriptively. For categorical variables, absolute and relative (%) frequencies are given. Distributions of continuous variables were checked for normality by visual inspection and using the Shapiro–Wilk test. Mean values and standard deviations (SDs) are reported for all continuous variables. In addition to the detailed assessment of the GACB score, a dichotomous categorization was used to facilitate a more distinct differentiation between patients with a GACB = 0, indicating no ACB, and those with a GACB ≥ 1, signifying the presence of anticholinergic stress. Group differences for continuous variables were tested using the Student's *t* test or Mann–Whitney *U* test; for categorical variables, Fisher's Exact Test was applied. Spearman's rank correlation coefficient was used to determine the associations between the number of chronic diseases, DMFT, PSI, age, ACB, USFR (mL/min), and xerostomia. This coefficient categorizes associations into five levels: very weak (0–< 0.2), weak (0.2–< 0.4), moderate (0.4–< 0.6), strong (0.6–< 0.8), and very strong (0.8–1). All reported *p*‐values are two‐sided and considered statistically significant if smaller than 5%; *p*‐values were not adjusted for multiplicity. All calculations were carried out using SPSS Statistics 29 64‐bit (IBM Corp., Armonk, NY, USA).

## 3 Results

### 3.1 Demographic and Clinical Characteristics

Data from 172 patients were included in the analysis [75 (43.6%) women, 97 (56.4%) men; mean age 65.67 (SD 9.51) years] (Table 1). The GACB score was 0 in 131 (76.2%) patients and ≥ 1 in 41 (23.8%) patients. The mean number of teeth was 23.52 (SD 5.61), with a difference (*p* = 0.005) between patients with GACB ≥ 1 [21.76 (SD 5.41) teeth] versusGACB = 0 [24.07 (SD 5.57) teeth]. There was an association between unstimulated hyposalivation and GACB; signs of unstimulated hyposalivation were evident in 25/41 (61.0%) patients with GACB ≥ 1 versus 9/131 (6.8%) patients with GACB = 0 (*p* < 0.001). The mean number of chronic systemic conditions was 1.81 (SD 2.02), with a difference (*p* < 0.001) between groups: an average of 3.71 (SD 2.44) chronic diseases were reported in patients with GACB ≥ 1 (*n* = 41) compared with 1.21 (SD 1.43) in patients with GACB = 0 (*n* = 131). The most common chronic systemic conditions were cardiovascular diseases (*n* = 70, 40.7%), thyroid diseases (*n* = 30, 17.5%), diabetes (*n* = 21, 12.2%), and depression (*n *= 9, 5,2%). For the most common chronic diseases, there was a significant difference in frequency between the GACB = 0 and GACB ≥ 1 groups (all *p* < 0.05). Twenty‐three (13.4%) patients had polypharmacy, defined as ≥ 5 medications. Of these, 4 (17.4%) took no anticholinergics, 8 (34.8%) took one anticholinergic, and 11 (47.8%) took two anticholinergics. There was considerable heterogeneity in the medications taken, which were based on their individual disease situation (e.g., frequent use of citalopram and mirtazapine as exemplary anticholinergics for depression). The most common drugs were acetylsalicylic acid, atorvastatin, and candesartan. Metformin was the most common anticholinergic used, scoring 1 (Table 2).

**Table 1:** Clinical characteristics (SD = standard deviation).

	Patients (*N* = 172)	ACB = 0 (*n* = 131)	“ACB ≥ 1” *(n* = 41)	*p*‐Value^a^
	Number of patients, *n* (%)			
Gender, female	75 (43.6)	54 (41.2)	21 (51.2)	0.280
Unstimulated hyposalivation	34 (19.8)	9 (6.8)	25 (61.0)	**< 0.001**
ACB				
0	131 (76.2)	131 (100)	—	
1	25 (14.5)	—	25 (61.0)	
2	14 (8.1)	—	14 (34.1)	
3	2 (1.2)	—	2 (4.9)	
Anticholinergic medication				
0	130 (75.6)	131 (100)	—	**< 0.001**
1	27 (15.8)	—	27 (65.9)	**< 0.001**
2	14 (8.2)	—	14 (34.1)	**< 0.001**
	Mean (SD)			
Age, years (*n* = 172)	65.67 (9.51)	64.37 (9.07)	69.83 (9.79)	**0.002**
No. teeth (*n* = 172)	23.52 (5.6)	24.07 (5.57)	21.76 (5.40)	**0.005**
PSI (*n* = 168)	2.82 (0.81)	2.81 (0.84)	2.85 (0.73)	0.810
Medications (*n* = 171)	2.01 (2.66)	1.04 (1.43)	5.07 (3.29)	**< 0.001**
Chronic systemic diseases (*n* = 171)	1,81 (2.02)	1.21 (1.43)	3.71 (2.44)	**< 0.001**
DMFT (*n* = 172)	17.88 (5.04)	17.79 (4.99)	18.15 (5.24)	0.540
XVAS (*n* = 172)	3.26 (2.72)	2.92 (2.66)	4.34 (2.65)	**0.003**
USFR (mL/min, *n* = 172)	0.41 (0.31)	0.48 (0.32)	0.20 (0.19)	**< 0.001**
ACB	0.34 (0.68)	—	1.44 (0.59)	

Abbreviations: ACB, anticholinergic burden; DMFT, Decayed Missed Filled Teeth; PSI, periodontal screening index; USFR, unstimulated salivary flow rate; XVAS, xerostomic visual analog scale.

^a^ *p* < 0.05 indicates statistical significance between “ACB = 0” and “ACB ≥ 1” patients (bold numbers).

**Table 2:** Top 20 most common drugs used by patients.

	Drug	Application area	Frequency	GACB score
1	Acetylsalicylic acid	Pain relief, anti‐inflammatory, and blood thinner	37/172	0
2	Atorvastatin	Cholesterol management and cardiovascular disease prevention	32/172	0
3	Candesartan	Hypertension and heart failure	26/172	0
4	l‐Thyroxine	Hypothyroidism	26/172	0
5	Ramipril	Hypertension and heart failure	23/172	0
6	Simvastatin	Cholesterol management	21/172	0
7	Bisoprolol	Hypertension and heart issues	16/172	0
8	Ibuprofen	Pain relief and anti‐inflammatory	14/172	0
9	Torasemide	Edema associated with heart failure and liver disease	14/172	0
10	Pantoprazole	Acid reflux and stomach ulcers	12/172	0
11	Allopurinol	Gout and kidney stones	11/172	0
12	Ezetimibe	Cholesterol management	11/172	0
13	Metformin	Type 2 diabetes management	10/172	1
14	Enalapril	Hypertension and heart failure	10/172	0
15	Cetirizine	Allergies	9/172	1
16	Metoprolol	Hypertension and heart issues	8/172	0
17	Omeprazole	Acid reflux and stomach ulcers	8/172	0
18	Bisoprolol	Hypertension and heart issues	8/172	0
19	Clopidrogel	Antiplatelet medication	7/172	0
20	Citalopram	Depression and anxiety disorders	7/172	1

Abbreviation: GACB, German anticholinergic burden (Kiesel, Hopf, and Drey 2018).

### 3.2 Influence of Age

Age‐classified descriptive data for clinical and dental characteristics, GACB, and subjective and objective dry mouth scores are shown in Table 3. The mean number of prescribed medications and number of chronic systemic diseases were higher in the older age groups (*p* < 0.001). Patients aged 76–80 years had the highest mean number of prescribed medications [4.13 (SD 3.20)] and GACB score [0.62 (SD 0.77)]. Age‐related differences were also evident for oral health: the DMFT score was higher in the older age groups (*p* = 0.007) and highest in the age group 71–75 years [20.15 (SD 4.49)]. No significant age differences were observed for perceived xerostomia and unstimulated salivation. The number of anticholinergic drugs was higher with age but did not reach statistical significance (*p* = 0.052).

**Table 3:** Descriptive age‐dependent numbers of clinical characteristics, ACB, and subjective and objective dry mouth values (SD = standard deviation).

	Patients (*N* = 172)	Age, years						*p*‐Value^a^
		50–55 (*n* = 27)	56–60 (*n* = 35)	61–70 (*n* = 55)	71–75 (*n* = 27)	76–80 (*n* = 15)	> 80 (*n* = 13)	
	Number of patients, *n* (%)							
Gender, female	75 (43.6)	9 (33.3)	12 (34.3)	25 (45.5)	14 (51.9)	8 (53.3)	7 (53.8)	0.51
	Mean (SD)							
Age (years, *n* = 172)	65.67 (9.51)	53.37 (1.39)	57.91 (1.46)	65.05 (2.77)	72.93 (1.21)	78.73 (1.16)	84.54 (1.85)	
Total API (*n* = 171)	2.01 (2.66)	1.07 (2.77)	1.00 (1.23)	1.85 (2.02)	2.89 (3.36)	4.13 (3.20)	2.92 (3.38)	**< 0.001**
Chronic systemic diseases (*n* = 171)	120 (69.8)	0.85 (1.23)	1.06 (1.14)	1.58 (1.54)	3.15 (3.28)	3.33 (1.99)	2.31 (1.55)	**< 0.001**
ACB (*n* = 172)	0.32 (0.68)	0.11 (0.32)	0.23 (0.65)	0.33 (0.67)	0.52 (0.80)	0.53 (0.83)	0.62 (0.77)	0.069
DMFT (*n* = 172)	17.88 (5.04)	16.93 (4.57)	15.69 (5.24)	18.45 (5.27)	20.15 (4.49)	17.47 (4.82)	19.08 (3.50)	**0.007**
XVAS (*n* = 172)	3.26 (2.72)	2.57 (2.60)	3.11 (2.62)	3.23 (2.73)	3.55 (2.69)	4.78 (2.94)	2.85 (2.73)	0.290
USFR (mL/min)	0.41 (0.31)	0.44 (0.24)	0.48 (0.37)	0.40 (0.34)	0.35 (0.27)	0.39 (0.27)	0.40 (0.34)	0.490
Anticholinergic medications	0.32 (0.62)	0.11 (0.32)	0.17 (0.45)	0.29 (0.57)	0.52 (0.80)	0.53 (0.83)	0.62 (0.77)	**0.052**

Abbreviations: ACB, anticholinergic burden; DMFT, Decayed Missed Filled Teeth; USFR, unstimulated salivary flow rate; XVAS, xerostomic visual analog scale.

^a^ *p* < 0.05 indicates statistical significance (bold numbers), evaluated using Fisher's Exact Test or the Mann–Whitney *U* test.

### 3.3 Correlation Between GACB and Oral Health Parameters

Spearman's correlation values assessed the associations between clinical and dental characteristics, GACB, USFR, and xerostomia (VAS) across all patients (*n* = 172) (Table 4). A moderate negative correlation was found between the GACB and USFR ($\overset{®}{r}$ = −0.51). A weak positive correlation was evident between ACB and xerostomia ($\overset{®}{r}$ = 0.23). There was a positive correlation between age and both GACB ($\overset{®}{r}$ = 0.253) and the number of chronic diseases ($\overset{®}{r}$ = 0.54) (*p* < 0.001). There was a negative correlation between USFR and xerostomia ($\overset{®}{r}$ = −0.39, *p* < 0.001). For patients with a GACB score ≥ 1 (*n* = 41), a moderate positive correlation emerged between anticholinergic load and the number of chronic diseases ($\overset{®}{r}$ = 0.52). Furthermore, there was a negative correlation between UFSR and both xerostomia ($\overset{®}{r}$ = −0.35) and GACB ($\overset{®}{r}$ = −0.25).

**Table 4:** Spearman's correlation values between different clinical and dental characteristics, anticholinergic burden, unstimulated salivary flow rates (mL/min), and xerostomia (VAS) (SD = standard deviation).

	All patients (*N* = 172)		
Spearman's (*p*‐values^a^)	ACB score	USFR (mL/min)	Xerostomia (VAS)
ACB (yes or no)		**−0.51 (**< **0.001)**	**0.24 (0.002)**
USFR (mL/min)	**−0.513 (**< **0.001)**	—	**−0.389 (**< **0.001)**
XVAS	**0.238 (0.002)**	**−0.389 (**< **0.001)**	—
Age	**0.253 (**< **0.001)**	−0.14 (0.064)	**0.160 (0.036)**
Chronic systemic diseases	**0.540 (**< **0.001)**	**−0.27 (**< **0.001)**	0.15 (0.050)
DMFT	0.045 (0.562)	−0.06 (0.410)	0.04 (0.560)
PSI	**0.174 (0.040)**	−0.03 (0.680)	−0.05 (0.490)

	**Patients with ACB** ≥ **1 (*n*** = **41)**		
Spearman's (*p*‐values^a^)	ACB score	USFR (mL/min)	Xerostomia (VAS)
ACB (yes or no)	—	−0.25 (0.110)	0.21 (0.200)
USFR (mL/min)	−0.251 (0.113)	—	**−0.356 (0.022)**
XVAS	0.205 (0.197)	**−0.356 (0.022)**	—
Age	0.181 (0.256)	−0.24 (0.130)	0.17 (0.290)
Chronic systemic diseases	**0.523 (**< **0.001)**	−0.28 (0.08)	0.24 (0.130)
DMFT	−0.028 (0.861)	−0.05 (0.770)	−0.02 (0.890)
PSI	0.166 (0.363)	0.27 (0.090)	−0.09 (0.580)

Abbreviations: ACB, anticholinergic burden; DMFT, Decayed Missed Filled Teeth; PSI, periodontal screening index; USFR, unstimulated salivary flow rate; (X)VAS, (xerostomic) visual analog scale.

^a^ *p* < 0.05 indicates statistical significance (bold numbers).

## 4 Discussion

Our study was designed to assess whether the GACB score can be used as a risk assessment for dry mouth in dental practice. By summing the values for all of a patient's medications, the total GACB score can be calculated for each individual. A higher total score indicates a higher cumulative anticholinergic exposure. The GACB score is the first of its kind specifically developed for prescribing physicians in Germany and goes beyond a simple aggregation of existing scores. It re‐evaluates medications, especially those with discrepancies in their classification, and sensibly adjusts this list.

We have demonstrated a correlation between GACB, xerostomia, and hyposalivation. Almost a quarter of patients (23.8%) over the age of 50 years had a GACB score of ≥ 1, highlighting the prevalence of anticholinergic exposure in this age group. These findings are consistent with previous studies that have also found a significant correlation between anticholinergic exposure and dry mouth (Kersten et al. 2013; Rudolph, 2008; Tiisanoja et al. 2018). Our results suggest that a high GACB is associated with reduced unstimulated salivary flow and increased xerostomia, further supporting the prognostic validity of the tool for predicting xerostomia and hyposalivation. However, a direct comparison of these results with those of previous studies is not easily possible due to differences in study samples, methods of assessing ACB, and definitions and measurements of hyposalivation and xerostomia. Nonetheless, our findings support the hypothesis that higher ACB correlates with dry mouth, particularly in older age groups, where polypharmacy and related exposure to anticholinergic side effects are more prevalent (Stenbäck et al. 2023; Tiisanoja et al. 2020).

Contrary to previous findings (Tiisanoja et al. 2018), our study found that the USFR in patients without ACB remained consistently above 0.2 mL/min across all age groups, despite higher medication prescriptions and the prevalence of chronic diseases with age. This suggests that additional, not fully understood factors, such as compensatory biological mechanisms or the adaptability of salivary glands, may play a role (Vissink, Spijkervet, and Amerongen 1996). Thus, salivary glands might retain some functionality despite increasing GACB and potential risks to oral health. This maintenance of salivary production could be due to adaptive changes within the glands or the activation of alternative metabolic pathways unaffected by anticholinergic effects (Choi et al. 2013; Seagrave, Hildebrand, and Johnson 1996). Individual differences in sensitivity to anticholinergic medications, genetic factors, or overall health status could influence the maintenance of salivary flow rate. Notably, the number of medications in patients with a GACB score of ≥ 1 was significantly different, indicating that higher medication intake is closely linked to anticholinergic effects and the development of xerostomia. A positive GACB score of ≥ 1 was associated with unstimulated hyposalivation, fewer teeth, and more severe perceived xerostomia. Patients with clinical xerostomia and hyposalivation often had a GACB score of ≥ 1, underscoring the connection between exposure to anticholinergic medications and reduced salivary secretion as well as oral health issues. Previous research (Cheah et al. 2023) has established a link between xerostomia and high ACB scores. Our descriptive analysis showed that VAS scores for xerostomia increased with age, except in patients over 80 years of age, who reported lower perceived xerostomia. Patients over 80 years of age showed unique characteristics, such as lower medication intake and fewer chronic conditions, possibly indicating a survival bias—these individuals might be genetically robust or lead healthier lifestyles (Frese et al. 2020; Sekundo et al. 2020). This highlights the need for a nuanced consideration of this age group regarding the GACB.

In general, ACB scores (like the GACB) are crucial for dentists, as the increased risk of dry mouth affects oral hygiene adjustment and oral health‐related quality of life and may necessitate preventive measures (Arany et al. 2021; Tiisanoja et al. 2016). Managing ACB in dental practice offers substantial benefits for both short‐ and long‐term treatment outcomes and patient quality of life. In the short term, managing ACB can reduce side effects like dry mouth and physical discomfort, leading to better adherence to dental treatments. This is particularly important for the dental care of older patients who often take multiple medications. In the long term, reducing ACB can lower the risk of dementia and falls, improve overall health, and reduce hospitalizations (Fox et al. 2011). Effective implementation in dental practice requires comprehensive medication management, continuous training of dental staff, and interdisciplinary collaboration. Dentists should regularly review their patients' medication lists and use tools like the GACB scale to identify and quantify medications with anticholinergic properties. Close collaboration with pharmacists, geriatricians, and other healthcare providers is essential to minimize ACB by jointly seeking alternative medications with lower anticholinergic effects. Moreover, it is crucial to educate patients and their caregivers about the risks associated with high anticholinergic cognitive burden scores and the importance of adhering to prescribed medication regimens. Lifestyle changes, such as cognitive training and physical exercise, can also help mitigate the effects of anticholinergic medications. Through proactive management, dentists can significantly and sustainably improve their patients' well‐being.

Inherent limitations and potential biases of our study must be considered. The study sample consisted exclusively of patients from Germany, and the results were based on the new GACB scoring system (Kiesel, Hopf, and Drey 2018); therefore, the generalizability of our findings to populations in other countries may be limited. Like all ACB scales, the GACB scale tends to oversimplify pharmacological mechanisms and assumes that the anticholinergic effects of various medications add up linearly. This could impair the scale's validity (Mayer, Haefeli, and Seidling 2015; Villalba‐Moreno et al. 2016). Additionally, there is a possibility that factors that were unaccounted for such as diet, alcohol consumption, or conditions like anxiety and stress, which are associated with hyposalivation or xerostomia, may have introduced bias. Smoking and alcohol consumption, as lifestyle modifiers, exacerbate the cognitive risks associated with anticholinergic medications, with smoking altering drug metabolism and alcohol enhancing neurotoxic effects (Reinold et al. 2021). Furthermore, xerostomia was assessed using a single‐point VAS, which could potentially underestimate the severity of xerostomia compared to more comprehensive questionnaires like the Xerostomia Inventory (XI) (Thomson 2015). The USFR was measured for only 5 min during student courses due to practical time constraints, which, while not meeting all literature‐recommended standards (Navazesh 1993), has been used in more recent studies (Stenbäck et al. 2023). Limitations also arose from the study design, as it was a retrospective analysis, and sample size calculations were not performed. Additionally, it was not possible to assess inter‐examiner concordance or the reproducibility of results among examiners, as oral examinations were conducted only once. However, consistency of measurements was ensured through prior calibration of dentists across all student classes in the department. Future studies should compare the GACB score with existing ACB scores and further investigate the synergistic lifestyle modifiers of patients and their impact.

## 5 Conclusion

Our retrospective analysis suggests that the GACB score could be an efficient and useful tool to quickly and reliably assess the ACB of patients. We believe that the GACB score should be routinely assessed in patients aged 50 years and older to identify and adequately address the risks associated with higher GACB at an early stage. This proactive approach makes it possible to identify vulnerable patients, inform them about the possible consequences of hyposalivation and xerostomia, and initiate preventive measures to prevent oral diseases in the context of dry mouth.

## Author Contributions

C.M.S. was responsible for data generation and interpretation and drafted the manuscript. I.D. was responsible for data generation and reviewed the manuscript. M.J.W. contributed ideas for the study and was responsible for data analysis and review of the manuscript. A.G.B. conceived and designed the study, was responsible for the planning and conduct of the study, and reviewed the manuscript. All authors revised and approved the final manuscript.

## Ethics Statement

Study approval was obtained from the Ethics Committee of the University of Cologne, Germany (23‐1062‐retro, approval date 06‐02‐2023).

## Consent

As this study involved a retrospective analysis of existing medical records, collected during routine dental care, individual patient consent was deemed unnecessary by the Ethics Committee.

## Conflicts of Interest

The authors declare no conflicts of interest.

## Supporting information

Supporting information.

## Data Availability

Data are available upon request.
